# The Influence of Zinc Oxide Nanoparticles on Dispersion, Rheology, and Mechanical Properties of Epoxy-Based Composites

**DOI:** 10.3390/polym17243253

**Published:** 2025-12-06

**Authors:** Tsz Ting Wong, Solange Amigues, Firas Awaja

**Affiliations:** 1 Engmat, Ltd., H91 TK33 Galway, Ireland; 2 School of Medicine, University of Galway, H91 TK33 Galway, Ireland

**Keywords:** nanoparticles, epoxy-based composites, zinc oxide (ZnO), dispersion, rheological behaviour, mechanical properties, stress transfer

## Abstract

The impact of zinc oxide (ZnO) nanoparticles on the dispersion, rheological behaviour, and mechanical properties of epoxy-based composites was investigated. Through experimental examinations, we found that 100 nm ZnO with a 4 wt.% content, when incorporated into epoxy, demonstrated homogeneous dispersion. Conversely, an increase in ZnO nanoparticle content led to particle agglomeration within the composite’s core. Rheology tests revealed that the 4 wt.% ZnO/epoxy mixture exhibited the lowest shear stress value, surpassing even the neat epoxy. Additionally, theoretical models were employed to evaluate the stress–strain properties of the ZnO/epoxy with the hollow glass fibre composite system. The study demonstrates the critical role of ZnO nanoparticle content in achieving dispersion and mechanical strength without the need for chemical solvents or surface modifications. Furthermore, variations in ZnO content within the composite resulted in a differing Young’s Modulus and UV absorbability, highlighting the importance of nanoparticle concentration in determining material properties. The study also delves into the effects of core diameter, length of hollow glass fibres (HGF), and adhesive layer thickness on stress transfer and strain deformation mechanisms within the composite system.

## 1. Introduction

Nanoparticle-filled polymers represent a groundbreaking class of materials that have garnered significant attention in recent years due to their unique and enhanced properties [1]. These polymers are infused with nanoparticles, typically ranging in size from 1 to 100 nanometers, which can include materials such as carbon nanotubes, graphene, silica, or metal oxides. The incorporation of these nanoparticles imparts remarkable mechanical, thermal, and electrical properties to the polymers, opening up a wide array of applications across various industries. These advanced materials find utility in fields such as aerospace, automotive, electronic, and biomedical engineering. One key aspect that underscores the significance of nanoparticle-filled polymers is the understanding of stress transfer within the composite structure. Stress transfer analysis is crucial in elucidating how the embedded nanoparticles interact with the polymer matrix, influencing the overall mechanical performance of the material. This knowledge is pivotal for designing and optimizing these composite materials for specific applications, ensuring their efficacy in real-world scenarios.

Nanoparticles, ranging in size from 1 to 100 nm, exhibit diverse elements, forms, and shapes, making them versatile candidates for integration into polymers or compounding with other elements. These nanostructures find applications in structural, medical, and electronic fields. Inorganic nanoparticles, in particular, are widely utilized to functionalize materials for advanced and diverse applications, owing to their homogeneous readiness and unique physical properties. In the context of this project, a 100 nm ZnO nanoparticle was chosen for its exceptional ultraviolet (UV) absorbability within the 100–400 nm range, effectively covering the entire spectrum of UVA, UVB, and UVC [2,3]. ZnO, as an inorganic particle, boasts a high exciton binding energy of 60 meV and a band gap of 3.37 eV [4], enabling efficient physical UV absorption. The UV energy entering the band gap of ZnO is dissipated into other forms, such as heat and light. Remarkably, despite its UV absorption capabilities, ZnO exhibits relatively high thermal stability [5].

Particle agglomeration is the inherent problem of particle dispersion in a polymer matrix, especially the particles in nano-size range [6]. This results in a dramatic reduction in the bonding property between particle and polymer and thus the structural properties of the material.

While extensive research has demonstrated the benefits of ZnO nanoparticles in epoxy matrices, achieving optimal dispersion remains a challenge, with many studies relying on chemical solvents [7,8] or complex surface modifications [9,10]. These methods can introduce process complexities and potential degradation pathways. A significant gap exists in understanding how nanoparticle concentration alone, without additives, can be optimized to balance dispersion, rheology, and mechanical performance. The novelty of this work lies in demonstrating that an optimal concentration (4 wt.%) of untreated ZnO nanoparticles can lead to homogeneous dispersion and favourable rheological behaviour in epoxy. Furthermore, we apply a detailed theoretical shear lag model to this composite system, providing predictive insights into the stress–strain behaviour as a function of nanoparticle content and other geometric parameters, thereby bridging the gap between experimental material formulation and theoretical performance prediction.

On the other hand, polymer degradation [11] is the inherent problem of polymer exposure under UV radiation. This induces dissolution and cracking [12] in both the outer- and near-surface of the polymers. The phenomena are more serious in pristine polymers when combined with thermal degradation, especially in a cyclic exposure [13,14]. The embedded nanoparticles become ready to release out of the degraded polymer [15,16,17] under physical attacks, such as scratching and weathering. Hence, the functional and structural properties of the bulk composite are reduced. Nonetheless, the literature also indicated no evidence showing the release of free fibrous carbon nanotube from host polymers even after prolonged UV exposure. Hollow glass fibre (HGF) is introduced for reinforcement with the embedded ZnO nanoparticle performing maximum physical UV absorbability.

The analysis of mechanical properties is crucial for the development of this structural fibre composite, especially to understand the stress–strain properties. Extensive research has been carried out to evaluate the mechanical properties of single fibre composite by using experimental, theoretical, and numerical methodologies. Experimental analyses include the single fibre pullout test [18,19,20,21,22,23], single fibre fragmentation test [24], single fibre pushout (indentation) test [25], single fibre microbond (microdebond) test [26], and microfatigue test [27]. Two major theoretical approaches that analyze the mechanism of fibre pullout are the fracture mechanics method [28,29,30,31,32] and shear lag theory [33,34,35,36,37], and they have been discussed in detail by Kim [38].

Fracture mechanics method is based on the energy criterion in terms of interfacial fracture toughness. It concerns obtaining information of the damage process of fibre inside a composite, and a debond zone is considered as an interfacial crack. Single fibre fragmentation technique has been used to determine the effects of surface treatment on fibre strength because of its convenient and reproducible test method. Shear lag theory is based on the maximum shear stress criterion in terms of interfacial shear stress. It concerns obtaining information of the maximum shear and axial strength of fibre and other elements inside a composite at the initiation of fibre debonding. A complete debonding is defined as the occurrence when the interfacial shear stress exceeds the interfacial shear bond strength.

Theoretical analysis gives fundamental and principal knowledge on a matter from a mathematical perspective. Although the calculation based on the maximum shear stress criterion gives the results of an ideal scenario, the shear lag approach enables one to demonstrate a complete failure condition of a fibre composite system. Moreover, all interconnected indicative parameters showing in our derived equation can be manipulated to analyze their effects on the fibre composite system. Most importantly, due to the fact that the stress transfer analysis of our single fibre composite system under fibre pullout action is difficult to perform experimentally while obtaining responsive results, in order to understand the mechanical properties of our engineered fibre composite system, the theoretical method is adopted for our analysis.

In the scope of this project, our aim is to investigate the impact of varying amounts of ZnO nanoparticles on the mechanical properties of the fibre composite system and validate the observed patterns in mechanical behaviour. This validation is crucial to establish consistency with the results obtained from the dispersion of nanoparticles and the rheological properties of the resin. Beyond the ZnO nanoparticle content within the core, other parameters affecting the mechanical properties of the fibre composite system are also under scrutiny. These include the hollowness, length, and thickness of the hollow glass fibre, as well as the adhesive strength and thickness of the adhesive layers. Our theoretical model, which has been detailed in a separate paper, encompasses all relevant parameters and delineates the relationships among the physical and mechanical properties of the elements within this fibre composite system.

## 2. Materials and Methods

In our experimental analysis, zinc oxide nanoparticles with a diameter of 100 nm (100 nm ZnO) in nodular shape were utilized to mix with epoxy resin, specifically Araldite GY250 and Hardener HY956 at a ratio of 5:1. This mixture was then infiltrated into hollow glass fibres (HGFs) with inner and outer diameters of 100 μm and 125 μm, respectively. Various contents of 100 nm ZnO were tested, including 2 wt.%, 4 wt.%, 5 wt.%, and 7 wt.%, while no surface treatment was conducted on the 100 nm ZnO prior to usage. For comparison purposes, neat epoxy-filled HGFs were employed.

To ensure thorough infiltration, a vacuum infiltration setup was constructed in-house to facilitate the penetration of different ZnO/epoxy mixtures into the HGFs. Following a 24 h curing process at ambient room temperature (23 ± 2 °C) and approximately 50% relative humidity, the resulting ZnO/epoxy/fibre composites were examined using a Scanning Electron Microscope (SEM) to assess the dispersion condition of the nanoparticles.

The rheological tests were conducted using a TA Instruments AR-G2 (TA Instruments Headquarters: 159 Lukens Drive, New Castle, DE 19720, USA ) rotational rheometer equipped with a 25 mm parallel plate geometry and a 1 mm gap, under isothermal conditions at 25 °C. The shear stress was measured as a function of a steadily increasing shear rate from 0.1 to 40 s^−1^. For SEM analysis, the cured HGFs filled with the ZnO/epoxy composite were cryo-fractured in liquid nitrogen to expose a clean cross-section. The fractured samples were then mounted on aluminum stubs using carbon tape and sputter-coated with a thin layer of gold (approximately 10 nm) to ensure conductivity before imaging with a [Zeiss EVO MA10, Carl Zeiss IQS Deutschland GmbH, Garching b. München, Germany] scanning electron microscope at an accelerating voltage of 20 kV.

**Figure 1:**
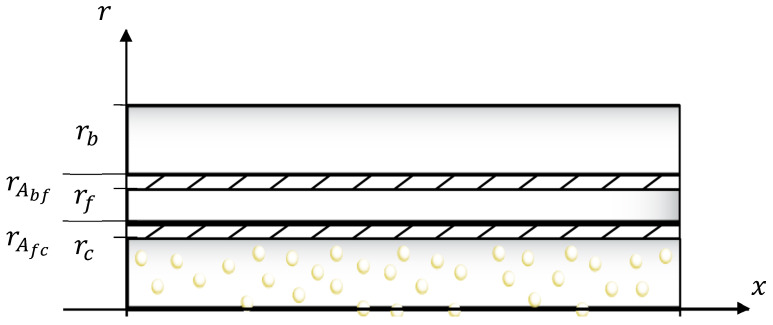
Five-cylinder model of ZnO/epoxy/fibre composite system for stress transfer analysis.

**Figure 2:**
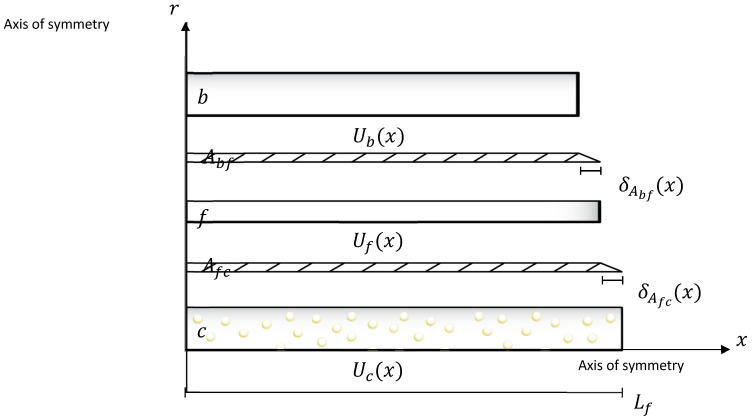
Axial displacement diagram of a ZnO/epoxy/fibre composite system.

These comprehensive analyses aim to elucidate the intricate relationships between nanoparticle content, dispersion, rheological behaviour, and stress transfer mechanisms within ZnO/epoxy/fibre composites, providing valuable insights for the optimization of such composite systems. In the stress transfer analysis, a five-cylinder model of the zinc oxide nanoparticle/mixed epoxy-filled/hollow glass fibre (ZnO/epoxy/fibre) composite system, illustrated in Figure 1, is proposed. The theoretical model employed here is based on a shear lag analysis, adapted for a multi-cylinder composite system representing the core, adhesive layers, and hollow fibre. The model assumes that the load is transferred between layers via interfacial shear stress. Equation (1), illustrated in Figure 2, describes the axial strain in the epoxy base (εb(x)) as a function of position x, derived from the force equilibrium equations of the system. In this equation, εc represents the maximum axial strain in the ZnO/epoxy core; Lf is the fibre length; and u and v are positive, real-valued parameters derived from the system of differential equations. These parameters are functions of the material properties (e.g., Young’s and shear moduli) and geometric dimensions (e.g., radii) of the constituent layers, encapsulating the efficiency of stress transfer through the interfaces.

(1) $$\varepsilon_{b}\left(x\right)=\varepsilon_{c}\left[1-\frac{vsinh\left(ux\right)-usinh\left(vx\right)}{vsinh\left(uL_{f}\right)-usinh(vL_{f})}\right]$$

$\varepsilon_{c}$ is the maximum axial strain of the ZnO/epoxy core; u and v are the real positive roots of $\tau_{A_{fc}c}\left(x,r_{c}\right)$ and $\tau_{A_{bf}f}\left(x,r_{f}\right)$, which are the shear stresses along the axial direction at the outer and inner interface of adhesive layer $A_{fc}$ and $A_{bf}$, respectively; and $L_{f}$ is the length of HGF. The theoretical model was developed to evaluate the stress–strain properties of this kind of fibre system and investigate the effects of the parameter change to this kind of fibre system.

## 3. Results and Discussion

Our work plan involved investigating how zinc oxide nanoparticles (100 nm ZnO) affect epoxy-based composites within hollow glass fibres (HGFs). We mixed Araldite GY250 and Hardener HY956 at a 5:1 ratio, adding varying 100 nm ZnO concentrations (2 wt.% to 7 wt.%) and neat epoxy for comparison. After vacuum infiltration into HGFs with 100 μm inner and 125 μm outer diameters, we cured the samples for 24 h. Then, we used a SEM to examine nanoparticle dispersion, assess rheological properties via ultrasonication and rotational rheometer testing, and develop a theoretical model to analyze stress transfer mechanisms. Our aim was to understand how nanoparticle content affects epoxy composite performance and optimize it for diverse applications.

**Figure 3:**
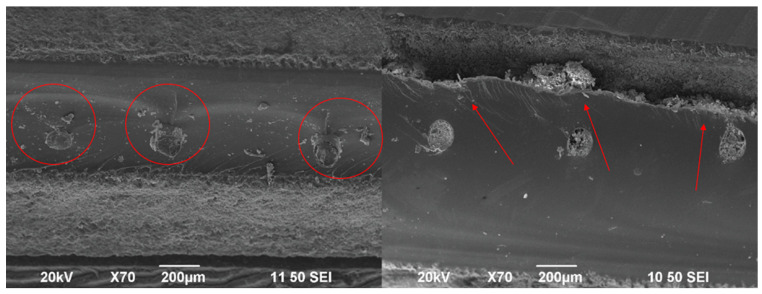
SEM images of a 4 wt.% (**left**) and 7 wt.% (**right**) ZnO/epoxy/fibre composite layer.

**Figure 4:**
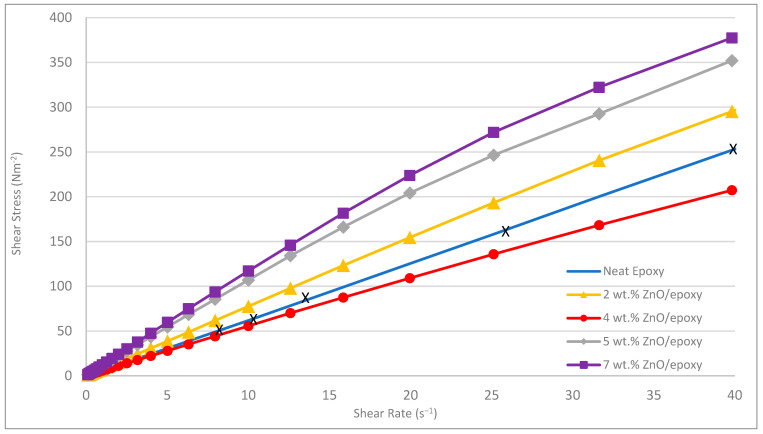
Shear stress under the increasing shear rate of different ZnO/epoxy resins.

Through experimental examinations, we discovered that the 100 nm ZnO with a 4 wt.% content, when mixed with epoxy, appeared to exhibit a homogeneous dispersion as shown in Figure 3. Particle agglomeration occurred with an increase in the content of ZnO nanoparticles in the core. A rheology test was conducted on the ZnO/epoxy fluid to examine the effects of nanoparticle content. Interestingly, the 4 wt.% ZnO/epoxy mixture exhibited a lower shear stress than the neat epoxy across the tested shear rates. This behaviour, while counter-intuitive, has been reported in other nanoparticle-filled polymer systems [39,40]. It is hypothesized that at this optimal concentration, the well-dispersed nanoparticles may disrupt intermolecular friction and polymer chain entanglements, effectively acting as a ‘lubricant’ and reducing the bulk viscosity [41]. At higher concentrations (5 and 7 wt.%), particle agglomeration leads to increased internal friction, resulting in the expected increase in shear stress, which is shown in Figure 4 [41].

From these observations, it is evident that the content of ZnO nanoparticles, without the aid of any chemical solvent or surface modification, plays a critical role in achieving homogeneous dispersion, efficient infiltration, and the desirable mechanical strength of the composite material [42].

While these SEM images provide a qualitative indication of improved dispersion at 4 wt.%, a rigorous quantitative assessment is necessary to confirm these findings. Future work should include a statistical analysis of agglomerate sizes from multiple micrographs and employ complementary techniques like Dynamic Light Scattering (DLS) on pre-cured suspensions to provide a more comprehensive understanding of the nanoparticle dispersion state. Furthermore, while SEM images provide a good overview of the micro-scale dispersion, we acknowledge that TEM would be required for a definitive nano-scale assessment of individual particle dispersion, which we propose for future work.

The stress–strain properties of the single ZnO/epoxy/fibre composite system were evaluated using the theoretical model [Equation (1)]. The theoretical formula yields six conditions for discussing the effects of parameter changes (E_c, r_c, L_f, t_f, G_A, and t_A) on the strain deformation of the epoxy base during the pullout of the ZnO/epoxy core.

Table 1 presents the geometrical and mechanical properties of the fibre composite system in a fundamental configuration, serving as the basis for our experimental analysis.

### 3.1. Content of ZnO Nanoparticles in the Core

The motivation for incorporating ZnO nanoparticles is their well-established ability to absorb UV radiation [1,2]. Different contents of ZnO nanoparticles are therefore expected to impart varying degrees of UV protection to the composite, in addition to influencing its Young’s Modulus. Utilizing the Rule of Mixtures (ROM) and the Voigt Model [43,44] (E_c = E_m V_m + E_p V_p) as a first-order approximation, we calculated the Young’s Modulus for composites and we calculated the Young’s Modulus for composites with different ZnO nanoparticle contents. The Young’s Modulus values for the neat composite, 2 wt.%, 4 wt.%, 5 wt.%, and 7 wt.% of ZnO/epoxy (E_(0%), E_(2%), E_(4%), E_(5%), and E_(7%)) are 3 GPa, 5.2 GPa, 7.3 GPa, 8.4 GPa, and 10.6 GPa, respectively. We acknowledge that the ROM assumes perfect bonding and dispersion, and therefore the calculated moduli for the 5 wt.% and 7 wt.% samples, where agglomeration was observed, likely represent an overestimation of the actual effective modulus.

Different Young’s Modulus of ZnO/epoxy core results in varied stress transfer and strain deformation mechanisms from the core to the base during fibre pullout. A higher content of ZnO nanoparticles in the epoxy core increases the stiffness of the ZnO/epoxy core, consistent with findings in other research papers [45,46,47,48]. The higher stiffness of the core allows it to absorb more stress per unit length, mitigating stress upon reaching the base and resulting in less strain deformation in the base.

**Figure 5:**
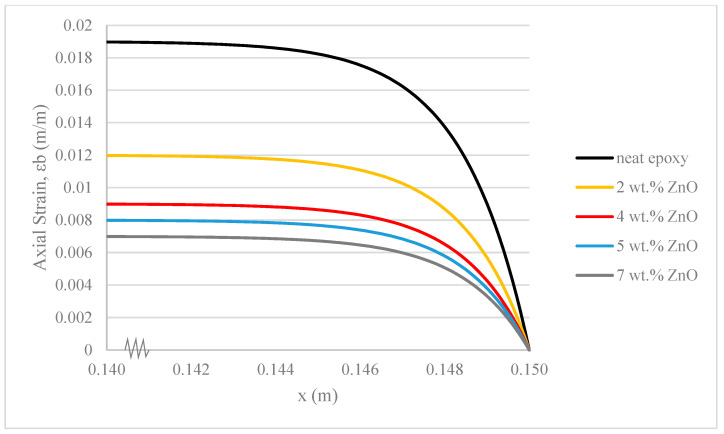
Strain deformation of epoxy base under fibre pullout with different contents of ZnO nanoparticles in core.

Figure 5 illustrates that the rate of change in strain deformation in the epoxy base in the composite with 4 wt.% ZnO in the epoxy core is significantly higher, and the strain deformation in the epoxy base is 50% lower than that with a neat epoxy core. Previous experimental analyses have shown that the 4 wt.% ZnO/epoxy/fibre composite achieves the highest UV absorbability among samples with 2 wt.%, 5 wt.%, and 7 wt.% ZnO nanoparticles. For epoxy resin with ZnO content higher than 4 wt.%, particle agglomerations were found inside the HGF [23]. Once particle agglomeration occurs, ZnO/epoxy inside the HGF becomes inhomogeneous, deviating from the theoretical analysis predicted in Figure 5. Its rate of change in strain deformation becomes slower, and the strain deformation of its epoxy base becomes higher.

For the theoretical analysis of stress transfer, a five-cylinder model is proposed to simulate the conditions during a fibre pullout event. It is important to note that physical pullout tests were not conducted in this study; rather, the model was developed to predict the stress–strain properties of the system. In the theoretical analysis, the 4 wt.% ZnO/epoxy/fibre composite demonstrates satisfactory stress transfer capability, exhibiting less variation compared to 5 wt.% and 7 wt.% ZnO, while showing more deviation from neat epoxy and 2 wt.% ZnO in terms of strain deformation. Therefore, the 4 wt.% ZnO/epoxy/fibre composite is considered as a reference sample for the subsequent analysis.

### 3.2. Hollowness of Hollow Glass Fibres

Different levels of hollowness in HGF ($r_{c})$ imply varying diameters of the ZnO/epoxy core. While keeping the parameter settings for material properties and geometrical factors of other elements constant within the composite system, we examined the effects of core diameters—45 μm, 63 μm, 81 μm, 90 μm, and 117 μm, each with a 1% adhesive layer—inside HGF with outer diameters of 104 μm, 122 μm, 140 μm, 149 μm, and 176 μm, respectively. The corresponding hollowness values for the HGF are 67%, 74%, 78%, 80%, and 84%.

A higher hollowness in HGF corresponds to a larger diameter of the ZnO/epoxy core. With the same ZnO nanoparticle content in epoxy, a larger core diameter implies lower stress tolerance per unit area and less deformation strain. Part of the stress transferred from the core to the base is transmitted to the surrounding structure.

**Figure 6:**
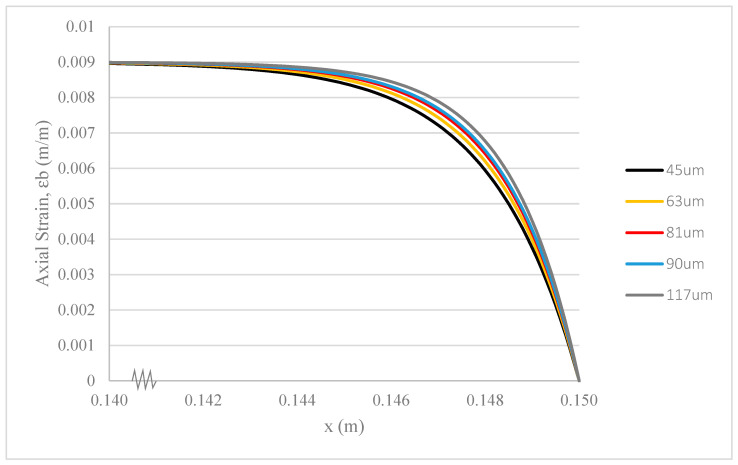
Strain deformation of epoxy base under fibre pullout with different hollowness of HGF.

Figure 6 is significant because it illustrates the trade-off between the core diameter and stress transfer. While a larger core (higher hollowness) experiences less deformation, indicating efficient stress transfer, the effect diminishes beyond a certain point (90 µm in our model). This analysis is crucial for design, as it helps identify an optimal HGF geometry that maximizes the load-bearing capacity of the core without being unnecessarily large, thus guiding the selection of fibre dimensions for practical applications. Although there is a small variation between the rates of change in strain deformation for different composites, the 4 wt.% ZnO/epoxy/fibre composite system with a ZnO/epoxy core diameter of 90 μm, HGF hollowness of 80%, and a 1% adhesive layer provides satisfactory stress transfer capability. This configuration is considered for experimental testing.

### 3.3. Length of Hollow Glass Fibres

The length of HGF ($L_{f}$) is a crucial consideration in configuring fibre composites, particularly in terms of stress transfer capability [34]. In general, for a unidirectional fibre composite, the longer the fibre, the more efficiently stress is transmitted to the fibre centre. Consequently, a composite reinforced by continuous fibres is superior to one with short fibres in bearing loads.

**Figure 7:**
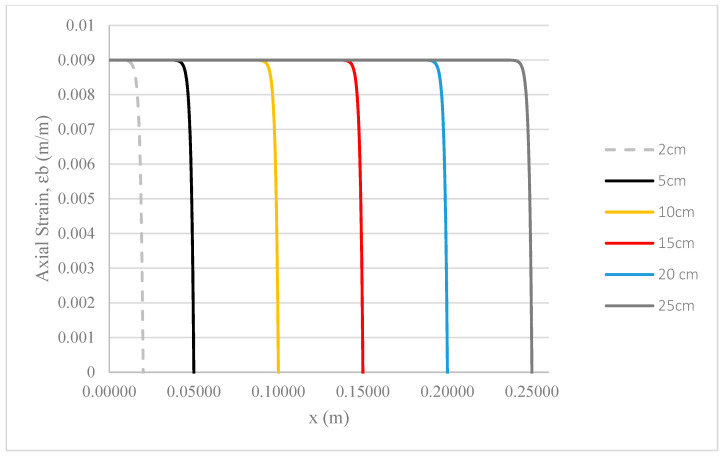
Strain deformation of epoxy base under fibre pullout with different lengths of HGF.

As shown in Figure 7, the rate of change in strain deformation in the epoxy base is consistent across composites with different lengths of HGF. The critical factor is the fibre’s length for load-bearing capacity. If the fibre length falls below a critical length, some of the load is transmitted to the epoxy, which has lower stiffness, thereby reducing the Young’s Modulus of the entire composite [25]. However, under the same applied stress for pulling out the fibre, all composites with different lengths of HGF analyzed can fully bear the specified loading and transfer stress to the epoxy base. The critical fibre length for the composite to handle the maximum loading is determined to be 2 cm. Therefore, a fibre with a length longer than its critical length under specific loading is sufficient for load-bearing, and in real-world applications, factors such as fibre continuity, orientation, and distribution are crucial parameters for determining the stress transfer capability of a fibre composite system [34].

### 3.4. Thickness of Hollow Glass Fibres

Different thicknesses of HGF ($t_{f})$ would alter its mechanical strength. With the same material properties and geometrical factors for other elements within the composite system during fibre pullout, the larger the thickness of HGF, the higher its tensile strength. This is due to a larger surface area perpendicular to the applied load, facilitating the transmission of stress per unit length [23].

**Figure 8:**
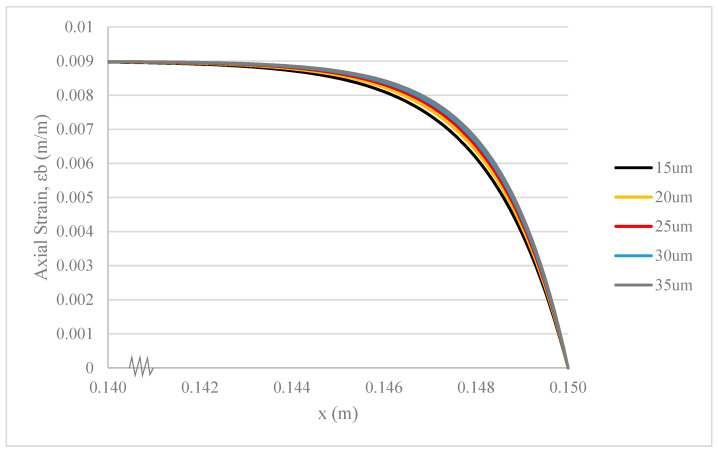
Strain deformation of epoxy base under fibre pullout with different thicknesses of HGF.

As shown in Figure 8, there is an imperceptible change in the rate of strain deformation in the epoxy base within the composite with different thicknesses of HGF. The small difference adequately indicates that the thickness of HGF is not a determining parameter for altering the stress transfer capability of the composite system [34]. A composite system with an HGF thickness of 25 μm is chosen as the configuration for experimental testing.

### 3.5. Shear Strength of Adhesive Layer and Thickness of Adhesive Layer

The shear strength ${(G}_{A})$ and the thickness of the adhesive layer ($t_{A})$ influence its shearing mechanism and influence the stress transfer and strain deformation conditions from layer to layer. The deformation of the adhesive layer under tensile loading is considered as being dominated by shear stress. According to the mathematical formula developed [u** and v**], the constant parameters of v and u are linear functions of the material properties, G, and geometrical factors, $\ln\left(\frac{r_{A_{fc}}}{r_{c}}\right)\;and\;\ln\left(\frac{r_{A_{bf}}}{r_{f}}\right)$, of adhesive layers, and they are combined into a linear sine function for the strain deformation of epoxy base, $\varepsilon_{b}\left(x\right)\;$[34]. And either a change in shear strength, G, or thickness,$\;r_{A_{fc}}-r_{c}\;and\;r_{A_{bf}}-r_{f}$, of adhesive layers results in a linear change to the strain deformation of the epoxy base [20].

The shear strength of adhesive layers can be modified by employing different coupling agents with distinct chemical formulas [18]. In common practice, a silane coupling agent is utilized to enhance the compatibility between epoxy and glass fibre. The shear strength of this adhesive layer is 1.2 GPa, and typically, silane (usually 1%) is added to the glass fibre surface to achieve favourable mechanical properties in fibre composites [49,50].

When altering the thickness of the adhesive layer to values of 2.8 μm, 3.5 μm, 4.0 μm, 4.5 μm, and 4.9 μm while maintaining a constant ZnO/epoxy core diameter of 90 μm, the weight percentages of the silane coupling agent in the core become 0.4%, 0.6%, 0.8%, 1%, and 1.2%, respectively.

**Figure 9:**
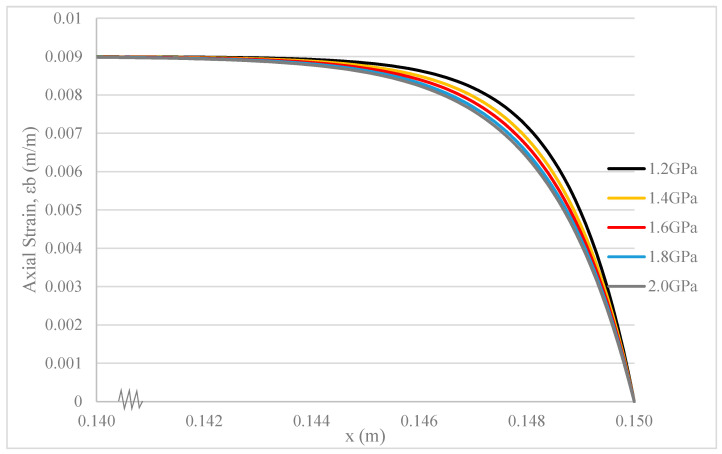
Strain deformation of epoxy base under fibre pullout with different shear strengths of adhesive layers.

As illustrated by Figure 9, the strain deformation in the epoxy base of the composite with adhesive layers of different shear strengths or thicknesses occurs at almost the same rate. However, a noticeable difference appears when comparing this rate to that of the composite with an adhesive layer possessing a shear modulus of 1.2 GPa and a thickness of 2.8 μm. A higher rate of strain deformation in the epoxy base is observed in composites with adhesive layers of lower shear strength or lesser thickness [19,20].

As the shear modulus of the adhesive layer decreases, its shear deformation increases. Similarly, as the thickness of the adhesive layer decreases, its shear deformation also increases. These phenomena occur under conditions where the ultimate shear strength of the adhesive layer is not surpassed. Consequently, less remaining stress is transferred to the epoxy base, leading to an increased rate of strain deformation. However, it is crucial to adopt an optimal shear modulus and thickness for the adhesive layer. Excessively low shear modulus or insufficient thickness of the adhesive layer can compromise the stress tolerance of the fibre composite, resulting in premature failure [18,23].

**Figure 10:**
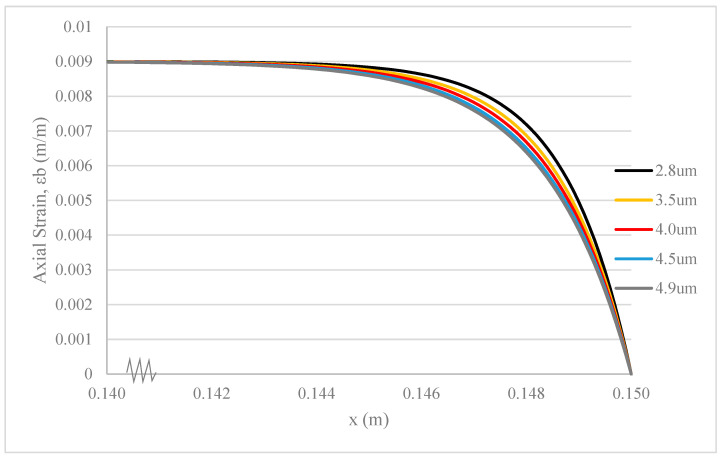
Strain deformation of epoxy base under fibre pullout with different thicknesses of adhesive layers.

There are similar curve shapes in Figure 8, Figure 9 and Figure 10. However, the key insight from comparing these figures lies in the relative sensitivity of the composite’s strain deformation to each parameter. Figure 8 shows that changes in HGF thickness (from 15 to 35 µm) have a nearly imperceptible effect on the base strain, indicating that the system’s stress transfer capability is robust and relatively insensitive to this parameter within the tested range. In contrast, Figure 9 and Figure 10 show that the system is significantly more sensitive to the properties of the thin adhesive layers. Even small changes in the adhesive shear modulus (Figure 9) or thickness (Figure 10) produce more noticeable shifts in the strain curve. This comparison highlights that for optimizing the mechanical performance of this composite, focusing on the interfacial adhesive properties is far more critical than on the HGF wall thickness.

## 4. Conclusions

In conclusion, this study provides a practical guideline for the formulation of epoxy-based composites with ZnO nanoparticles using a simple, solvent-free method. Our findings demonstrate that a concentration of 4 wt.% offers an optimal balance between achieving homogeneous nanoparticle dispersion and favourable rheological properties, which our theoretical model predicts will translate to efficient stress transfer and enhanced stiffness. This is practically significant as it suggests that complex surface treatments or the use of chemical solvents may not be necessary to achieve desirable properties, simplifying the manufacturing process and reducing environmental impact. The presented theoretical framework further serves as a valuable tool for designing and optimizing similar hollow-fibre composite systems for specific loading applications. It is crucial to note that the conclusions regarding mechanical performance are based on the predictions of our theoretical model. The primary aim of this work was to establish a processing–performance framework by correlating fundamental experimental data (dispersion, rheology) with this theoretical model. Direct experimental validation of the mechanical properties, such as tensile and flexural testing, is a critical next step and the subject of our ongoing research. Such experiments will serve to validate the predictions made herein and provide a complete picture of the composite’s behaviour.

## Figures and Tables

**Table 1:** Geometrical and mechanical properties of the fibre composite system in a fundamental configuration.

Elements	Symbols	Values
Geometry		
Epoxy base	$r_{b}$	208 μm
Adhesive layer	$r_{A_{bf}}$	158 μm
HGF	$r_{f}$	149 μm
Adhesive layer	$r_{A_{fc}}$	99 μm
ZnO/epoxy core	$r_{c}$	90 μm
Young’s Modulus		
Epoxy	$E_{b}$	3 GPa
HGF	$E_{f}$	68.5 GPa
Adhesive layer	$E_{A}$	3.3 GPa
ZnO	$E_{ZnO}$	111.2 GPa
Adhesive Layers		
Adhesive layer	$G_{A}$	1.2 GPa
Thickness of adhesive layer	$t_{A}$	4.5 μm

## Data Availability

The original contributions presented in this study are included in the article. Further inquiries can be directed to the corresponding author.
